# Prognostic Value of Investigating Neuron-Specific Enolase in Patients with Ischemic Stroke

**DOI:** 10.17691/stm2021.13.2.08

**Published:** 2021-01-01

**Authors:** А.S. Kurakina, T.N. Semenova, E.V. Guzanova, V.N. Nesterova, N.A. Schelchkova, I.V. Mukhina, V.N. Grigoryeva

**Affiliations:** Assistant, Department of Neurology and Medical Genetics, Perm State Medical University named after Academician E.A. Wagner, 26 Petropavlovskaya St., Perm, 614990, Russia; Assistant, Department of Neurological Diseases, Privolzhsky Research Medical University, 10/1 Minin and Pozharsky Square, Nizhny Novgorod, 603005, Russia; Associate Professor, Department of Neurological Diseases, Privolzhsky Research Medical University, 10/1 Minin and Pozharsky Square, Nizhny Novgorod, 603005, Russia; Chief of Regional Vascular Center No.23; Head of the Unit for Patients with Acute Disorder of Cerebral Circulation, Nizhny Novgorod Regional Clinical Hospital named after N.A. Semashko, 190 Rodionova St., Nizhny Novgorod, 603126, Russia; Head of the Central Scientific Research Laboratory, Privolzhsky Research Medical University, 10/1 Minin and Pozharsky Square, Nizhny Novgorod, 603005, Russia; Associate Professor, Department of Normal Physiology named after N.Y. Belenkov, Privolzhsky Research Medical University, 10/1 Minin and Pozharsky Square, Nizhny Novgorod, 603005, Russia; Professor, Director of the Institute of Fundamental Medicine, Privolzhsky Research Medical University, 10/1 Minin and Pozharsky Square, Nizhny Novgorod, 603005, Russia; Head of the Department of Normal Physiology named after N.Y. Belenkov, Privolzhsky Research Medical University, 10/1 Minin and Pozharsky Square, Nizhny Novgorod, 603005, Russia; Professor, Head of the Department of Neurological Diseases, Privolzhsky Research Medical University, 10/1 Minin and Pozharsky Square, Nizhny Novgorod, 603005, Russia

**Keywords:** ischemic stroke, neuron-specific enolase, functional outcome predictors

## Abstract

**The aim of the study** was to assess the prognostic value of the plasma neuron-specific enolase (NSE) level as a predictor of functional outcome and motor function recovery in the acute period of ischemic stroke (IS).

**Materials and Methods:**

Fifty patients with IS have been examined. On admission to the hospital and at 12–14 days after stroke onset, a clinical and neurological examination have been carried out with the supplementary quantitative assessment of neurological deficit severity according to the National Institutes of Health Stroke Scale (NIHSS), functional outcome according to the Modified Rankin Scale, and Rivermead Mobility Index. Enzyme immunoassay was used to determine NSE concentration in blood plasma in the acute period of the disease.

**Results:**

The NSE level in patients’ blood plasma in the first 48 h after stroke onset positively correlates with the ischemic focus volume (r=0.49; p=0.003) and the severity of neurological symptoms (according to NIHSS) (r=0.33; p=0.02). NSE less than 2 ng/ml in the acute disease period is a predictor of good functional outcome 12–14 days after stroke onset (OR=12.4; р=0.006). The NSE level >2.6 ng/ml is associated with a high likelihood of lethal outcome.

Neurological deficit below 15 according to NIHSS as well as the NSE level <2 ng/ml in the acute IS period are estimated as prognostic factors of significant recovery of motor function at 2 weeks after disease onset (OR=5.8; р=0.02).

**Conclusion:**

Determination of NSE in blood plasma makes it possible to predict functional outcome of the disease development and the recovery of motor function in patients with IS.

## Introduction

Stroke is one of the leading causes of persistent loss of disability among the population [1]. In the majority of cases, this is connected with the development of motor deficit in this cohort of patients [2].

Improvements in rendering medical aid to patients with stroke are promoted by the search for laboratory biomarkers allowing one to define brain damage severity caused by acute ischemia and evaluate the prognosis of functional rehabilitation of these patients.

One of such markers may be neuron-specific enolase (NSE). It represents an intracellular enzyme of neurons and neuroendocrine cells [3].

Ischemic stroke (IS) results in neuron death, hematoencephalic barrier (HEB) impairment, and increase of NSE concentration in blood [4]. However, researchers have not arrived at a common view in relation to the diagnostic and prognostic value of the NSE level in the blood of patients with stroke [5–10].

**The aim of the study** was to assess the prognostic value of the plasma neuron-specific enolase level as a predictor of functional outcome and motor function recovery in the acute period of ischemic stroke.

## Materials and Methods

The examined patients were initially admitted for treatment to the Resuscitation Unit of Regional Vascular Center No.2 with the following transfer to the Neurological Department for Patients with Acute Disorders of Cerebral Circulation (ADCC) at Nizhny Novgorod Regional Clinical Hospital named after N.A. Semashko in 2019–2020 (Russia).

Patients were selected using the continuous sampling method. Criteria for participation in the study included age from 18 to 80 years, voluntary consent for participation, definite diagnosis of the first-time stroke, and the first 48 h after symptom development. Exclusion criteria were as follows: autoimmune diseases in the medical history, chronic kidney diseases (GFR<60 ml/min/1.73 m^2^), hyperthermia of inflammatory genesis, oncopathologies of extra-cerebral and cerebral location, traumatic brain injury during the previous three months, neurosurgical intervention, hemorrhagic stroke or IS with a hemorrhagic transformation, recurrent stroke.

The diagnosis of ischemic stroke was established on the basis of patient’s complaints, history data, findings of the neurological examination, CT, and MRT. Ischemic focus volume was estimated using 0.52**·***ABC* formula, where *А*, *В*, *С* are the main maximum dimensions of the ischemic focus measured in three projections.

Fifty patients with IS (27 (54±7%) women and 23 (46±7%) men) aged from 37 to 80 years (mean age — 66.5±10.4 years) participated in the study.

On admission to the hospital (Т_0_), all patients underwent clinical and neurological examination with a supplementary assessment of the consciousness impairment degree according to the Glasgow Coma Scale (GCS), the severity of neurological deficit using the National Institutes of Health Stroke Scale (NIHSS), functional outcome according to the Modified Rankin Scale (mRS), Rivermead Mobility Index, and NSE concentration in blood plasma determined by enzyme immunoassay method (Vector Best, Russia).

At 12–14 days after the stroke (Т_1_), the severity of neurological deficit using NIHSS, functional outcome according to mRS, and Rivermead Mobility Index were reassessed.

In terms of the Modified Rankin Scale, the score of 0–2 indicated good functional outcome while the score of 3–6 designated poor outcome [11].

Significant recovery of motor function was diagnosed at the increase of the Rivermead mobility index by more than 2 points, insignificant recovery/no recovery at its increase by 0–2, significant worsening — at the index decrease by more than 2 points [12].

The study complies with the Declaration of Helsinki (2013) and was approved by the Ethics Committee of Privolzhsky Research Medical University (Nizhny Novgorod, Russia). Written informed consent was obtained from each patient.

**The data were statistically analyzed** using a package of the applied programs Statistica 7.0 for Windows (StatSoft Inc., USA) and SPSS 17.0 (SPSS Inc., USA). Qualitative variables were checked for normality using the Shapiro–Wilk test. Mean (M) and standard deviation (σ) were calculated for the qualitative data that follow the normal distribution, while the median (Me) and interquartile range containing the 25^th^ and 75^th^ percentiles (Me [Q1; Q3]) were calculated for those data that were not distributed normally. To compare the two samples, the nonparametric Mann–Whitney test for independent groups was used. The interconnection of the parameters was assessed by the Spearman’s rank correlation method. Value differences between the groups were considered statistically significant at р<0.05. To determine the diagnostically significant cut-off point of NSE, a ROC analysis with a ROC curve building and indication of the area under the curve (AUC) was employed. The cut-off threshold was selected at the point of the plot having the highest total sensitivity and specificity. To evaluate separate factors affecting functional outcome in patients after IS onset, the odds ratio (OR) was calculated.

## Results

The acute period of IS (Т_0_) was characterized by the following clinical and neurological parameters (score according to different scales): GCS — 13.9±2.1; NIHSS — 10.2±7.3; Rivermead — 3.0 [0.0; 5.5]; mRS — 4.2±0.7; IS focus volume — 9.3 [0.8; 36.0] сm^3^; focus localization: supratentorial (n=40) — 80.0±5.7%, subtentorial (n=10) — 20.0±5.7%; essential hypertension (n=46) — 92.0±2.8%; dyslipidemia (n=18) — 36.0±6.8%; diabetes mellitus (n=10) — 20.0±5.7%; atrial fibrillation (n=10) — 20.0±5.7; NSE level — 2.0 [1.5; 3.0] ng/ml.

The NSE level in the patients’ blood plasma in the first 48 h after stroke correlated positively both with the ischemic focus volume (r=0.49, p=0.003) and the severity of neurological symptoms (NIHSS) (r=0.33, p=0.02). The intensity of neurological deficit (NIHSS), in its turn, was positively statistically significantly related to the volume of the ischemic focus (r=0.53, p=0.001).

Statistically significant correlations between the NSE level and the severity of functional impairment (according to the Rankin and Rivermead scales) and the severity of patient’s state according to the GCS have not been found in patients in the acute IS period (r=0.07, p=0.58; r=–0.23, p=0.12; r=–0.22, p=0.15).

The comparative analysis of the NSE level in patients with supra- and subtentorially located ischemic focus did not detect any statistically significant differences (р=0.4).

At 12–14 days after stroke, good functional outcome (score 0–2, mRS) was observed in 12 patients (24±6%), whereas poor outcome (score 3–6, mRS) was determined in 38 patients (76±6%). A lower mean NSE level (1.7 [1.4; 1.8] ng/ml) measured in the acute period was characteristic for patients with good functional outcome than for those with poor outcome (2.1 [1.7; 3.0] ng/ml) (р=0.04).

The ROC analysis method was used to define a diagnostically significant point of division for the NSE level allowing patients with good functional outcome to be distinguished from those with poor outcome. The area under the obtained ROC curve (AUC) equaled to 0.73 (95% CI 0.54–0.92; р=0.04) (see the Figure (а)).

**Figure:**
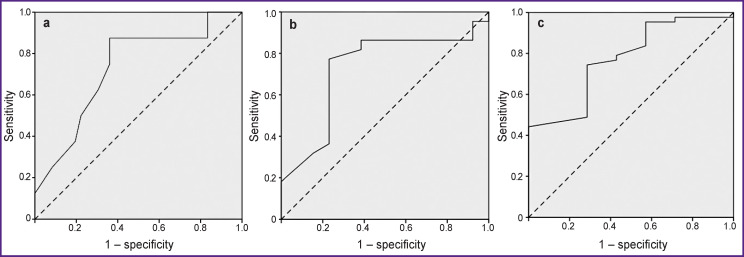
ROC curves of NSE (Т_0_) plasma level for prediction of functional outcome (а), motor function recovery (b), and lethal outcome (c) in patients 12–14 days after ischemic stroke onset

The maximum value of the sensitivity and specificity sum equal to 1.51 corresponded to the NSE concentration of 2 ng/ml (sensitivity of the method was 87.5%, specificity — 63.9%).

Determination of OR for functional outcome in patients at 12–14 days after IS development using different scales of the quantitative variables studied by us has shown that the NSE level in the acute period of the disease was of significance for the prognosis (Table 1).

**Table 1:** Odds ratio values for the development of good/poor functional stroke outcome (n=50)

Attribute	Attribute category	95% CI			p
		ОR	Low limit	Upper limit	
Patients’ age (years)	Below 61 (n=12) 61 and older (n=38)	2.3	0.2	23.1	0.42
Ischemic focus volume (сm^3^)	Less than 9.3 (n=22) 9.3 and more (n=28)	1.6	0.2	12.2	0.63
Stroke localization	Supratentorial (n=40) Subtentorial (n=10)	0.8	0.1	9.3	0.88
Thrombolysis	Performed (n=12) Not performed (n=38)	0.9	0.1	5.3	0.87
Assessment according to NIHSS (Т_0_) (score)	Less than 15 (n=36) 5 and more (n=14)	3.9	0.4	38.2	0.17
Assessment according to Rivermead (Т_0_) (points)	Less than 4 (n=30) 4 and more (n=20)	2.2	0.4	10.9	0.33
NSE concentration (Т_0_) (ng/ml)	Less than 2 (n=26) 2 and more (n=24)	12.4	1.3	119.7	**0.006**

At 12–14 days after IS onset, 25 patients (50.0±7.1%) were observed to recover significantly the motor function, in 15 patients (30.0±6.5%) there was insignificant or no regeneration, in 3 patients (6.0±3.4%) the motor function worsened significantly. Fatal outcome was observed in 7 patients (14.0±4.9%) within the first two weeks of IS.

The ROC analysis showed that the 2 ng/ml NSE (T_0_) level provides the possibility to identify patients with significant/insignificant recovery of motor function with 77.3% sensitivity and 76.9% specificity (AUC was 0.73; 95% CI 0.54–0.91; р=0.03) (see the Figure (b)).

The OR analysis of the significant motor function recovery in patients at 12–14 days after IS onset showed that the score of neurological deficit intensity below 15 was of value for the prognosis as well as the NSE level lower than 2 ng/ml in the first 48 h after disease onset (Table 2).

**Table 2:** Odds ratio values for significant/insignificant motor function recovery (n=40)

Attribute	Attribute category	95% CI			p
		ОR	Low limit	Upper limit	
Patients’ age (years)	Under 61 (n=8) 61 and older (n=32)	0.7	0.1	4.3	0.72
Ischemic focus volume (сm^3^)	Less than 9.3 (n=30) 9.3 and more (n=10)	2.4	0.4	14.7	0.32
Stroke localization	Supratentorial (n=32) Subtentorial (n=8)	0.9	0.1	6.9	0.94
Thrombolysis	Performed (n=11) Not performed (n=29)	0.6	0.1	2.7	0.49
Assessment according to NIHSS (Т_0_) (score)	Less than 15 (n=30) 15 and more (n=10)	5.4	1.1	29.6	**0.03**
Assessment according to Rankin (Т_0_) (points)	Less than 4 (n=5) 4 and more (n=35)	0.6	0.03	1.11	0.7
NSE concentration (Т_0_) (ng/ml)	Less than 2 (n=23) 2 and more (n=17)	5.8	1.2	29.3	**0.02**

The NSE (Т_0_) level appeared to be statistically significantly higher in patients who died within 14 days after IS development (n=7) than in the survived patients (3.0 [1.7; 6.0] and 1.9 [1.5; 2.6] ng/ml, respectively, p=0.02). A ROC point equal to 2.6 ng/ml was defined allowing the identification of the dead and survived patients with 74.7% sensitivity and 71.4% specificity (AUC was 0.77; 95% CI 0.60–0.95; р=0.02) (see the Figure (c)).

The increase of the NSE level above 2.6 ng/ml (OR=8.3; р=0.01) in the acute IS period is prognostically unfavorable in regard to the probability of lethal outcome.

## Discussion

NSE is a marker of damaged neurons since their death leads to the release of this enzyme to the extracellular medium which makes it possible to assess the extent of structural and functional impairment of the biomembranes in the central nervous system [13, 14].

According to some authors, NSE content in the blood is associated positively with the brain infarction volume [5, 6, 7, 9, 15], although there are works in which the interaction of this kind was not found [16]. Ambiguous is the opinion on the correlation between the NSE level and the severity of neurological symptoms in stroke: some researchers confirm this association [5, 6, 7, 15] while others deny it [8].

In our work, a positive statistically significant correlation has been shown between the NSE level, severity of neurological symptoms (according to NIHSS) in the acute period of the disease, and the volume of ischemic focus.

The obtained results may be explained by the fact that the larger the ischemic focus, the more substantial is the death of the neurons, resulting in greater HEB permeability. This process facilitates the NSE release to the peripheral bloodstream [3]. These processes are also reflected in the intensity of the neurological deficit in patients.

In the course of our study, the NSE content in blood plasma below 2 ng/ml in the acute IS period has been found to be associated with good functional outcome and significant recovery of motor function at 2–14 days after disease onset in more than 88% of cases.

A number of studies [6–8] have demonstrated that NSE levels in the blood of patients with acute disorders of cerebral circulation may be used to predict functional outcome at 1–3 months after the disease onset, but there are works asserting the opposite [5].

Prognostic value of NSE for motor function regeneration in patients with IS has not been previously studied.

The results of our investigation have demonstrated that the NSE level higher than 2.6 ng/ml in the acute IS period is a predictor of lethal outcome. In this connection, it should be noted that Ahmad et al. [15] and Thelin et al. [17] have found that high NSE level in patients with neurotrauma was associated with fatal outcome, though no correlations of this kind have been previously detected in IS patients.

## Conclusion

The intensity of neurological symptoms in patients with ischemic stroke and the ischemic focus volume correlate statistically significantly with the level of neuron-specific enolase in blood plasma which may be used for objectivization of patients’ state.

The NSE concentration below 2 ng/ml in the blood of patients in the acute period of ischemic stroke allows one to predict good functional outcome at 12–14 days after disease onset and along with the severity of neurological symptoms below the score of 15 (NIHSS) is a predictor of significant motor function recovery.
